# Four and a Half LIM Domains 2 (FHL2) Contribute to the Epithelial Ovarian Cancer Carcinogenesis

**DOI:** 10.3390/ijms21207751

**Published:** 2020-10-20

**Authors:** Chen Wang, Xiangmin Lv, Chunbo He, John S. Davis, Cheng Wang, Guohua Hua

**Affiliations:** 1 Key Lab of Agricultural Animal Genetics, Breeding and Reproduction of Ministry of Education, College of Animal Science and Technology, Huazhong Agricultural University, Wuhan 430070, China; chenwang0925@gmail.com; 2 Olson Center for Women’s Health, Department of Obstetrics and Gynecology, University of Nebraska Medical Center, Omaha, NE 68198-3255, USA; XLV@mgh.harvard.edu (X.L.); chunbo.he@unmc.edu (C.H.); jsdavis@unmc.edu (J.S.D.); cwang34@mgh.harvard.edu (C.W.); 3 Vincent Department of Obstetrics and Gynecology, Vincent Center for Reproductive Biology, Massachusetts General Hospital, Boston, MA 02114, USA; 4 Omaha Veterans Affairs Medical Center, Omaha, NE 68105, USA

**Keywords:** FHL2, epithelial ovarian cancer, cell growth, cell cycle, xenograft

## Abstract

Epithelial ovarian cancer (EOC) is one of the most lethal gynecologic malignancies. To date, the etiology of this deadly disease remains elusive. FHL2, a member of the four and a half LIM domain family, has been shown to serve either as an oncoprotein or as a tumor suppressor in various cancers. Our previous study showed that FHL2 plays a critical role in the initiation and progression of ovarian granulosa cell tumor via regulating AKT1 transcription. However, direct and systematic evidence of FHL2 in the initiation and progression of EOC remains unclear. In the present study, immunohistochemical analysis from EOC patient tissues showed that positivity and intensity of FHL2 immunosignal were up-regulated in the EOC tissues compared with normal ovary tissues. Knockdown of FHL2 in SKOV-3 cell line reduced cell growth and cell viability, blocked cell cycle progression, and inhibited cell migration. Ectopic expression of FHL2 in IGROV-1 cells which have low endogenous FHL2, promoted cell growth, improved cell viability and enhanced cell migration. Additionally, knock down of FHL2 in the SKOV-3 cell line significantly inhibited anchorage-independent growth indicated by the soft agar assay. In comparison, overexpression of FHL2 in IGROV-1 cell improved the colonies growth in soft agar. Western blot data showed that knockdown of FHL2 downregulated AKT expression level, and upregulated apoptosis related proteins such as cleaved PARP, and cleaved-lamin A. Finally, by employing stable SKOV-3/FHL2 stable knock down cell line, our data clearly showed that knockdown of FHL2 inhibited EOC xenograft initiation in vivo. Taken together, our results showed that FHL2, via regulating cell proliferation, cell cycle, and adhesion, has a critical role in regulating EOC initiation and progression. These results indicate that FHL2 could be a potential target for the therapeutic drugs against EOC.

## 1. Introduction

Ovarian cancer accounts for 2.5% of all malignancies among females and 5% of female cancer deaths because of its low survival rates, largely driven by late stage diagnoses. The American Cancer Society reported diagnosis of 22,240 new cases of ovarian cancer and 14,070 ovarian cancer deaths in the United States in 2018. Epithelial cancers, which is more aggressive than nonepithelial cancers, are most common among women of all racial/ethnic groups, accounting for 90% of all cases [1]. Epithelial ovarian cancers, which develop from transformation of the fallopian tube and/or ovarian surface epithelium (OSE) [2,3], are classified by tumor cell histology as serous (52%), endometrioid (10%), mucinous (6%), or clear cell (6%), with one-quarter classified as more rare or unspecified subtype [4].

Risk factors associated with ovarian epithelial cancer including race/ethnicity, age, genetic and reproductive/hormonal factors [5,6]. Mutations in many genes, including *TP53*, *BRCA1/2*, *PIK3CA*, *PTEN*, *CTNNB1* were implicated in genesis of the different types of EOC [4,7,8,9,10,11]. Yes-associated protein (YAP) interacts with ERBB signaling pathway to regulate the initiation and progression of EOC [12]. Higher positivity of estrogen (ER) or progesterone receptors (PR) was reported in high high-grade, low-grade serous and endometrioid carcinoma [13]. However, the etiology of EOC remains unclear. 

The four and a half LIM domains 2 (FHL2) is a multifunctional scaffolding protein regulating signaling cascades and gene transcription [14]. FHL2 can function as an oncoprotein or as a tumor suppressor in a tissue or cell type–dependent manner [15,16,17]. Our previous study showed that FHL2 plays a critical role in the initiation and progression of ovarian granulosa cell tumor (GCT) via controlling AKT1 gene transcription [18]. FHL2 protein expression is elevated in EOC tissues, suggesting an important functional role of in gynecologic malignancies [19]. However, further studies are necessary to provide more direct and systematic evidence on the role of FHL2 in the initiation and progression of EOC. In the present study, we showed that FHL2 is critical for EOC development. FHL2 may serve as a novel molecular target for EOC therapeutic drug development.

## 2. Results

### 2.1. FHL2 Is Overexpressed in Human EOC Tissues

In a previous study, we showed that FHL2 is overexpressed in the ovarian granulosa tumor cells [18]. To examine the FHL2 expression in the EOC tissues, immunochemistry was performed in EOC and normal ovary tissues. The immunochemistry staining revealed that FHL2 expression was significantly increased in tumor tissue when compared with normal ovarian tissue.

**Figure 1:**
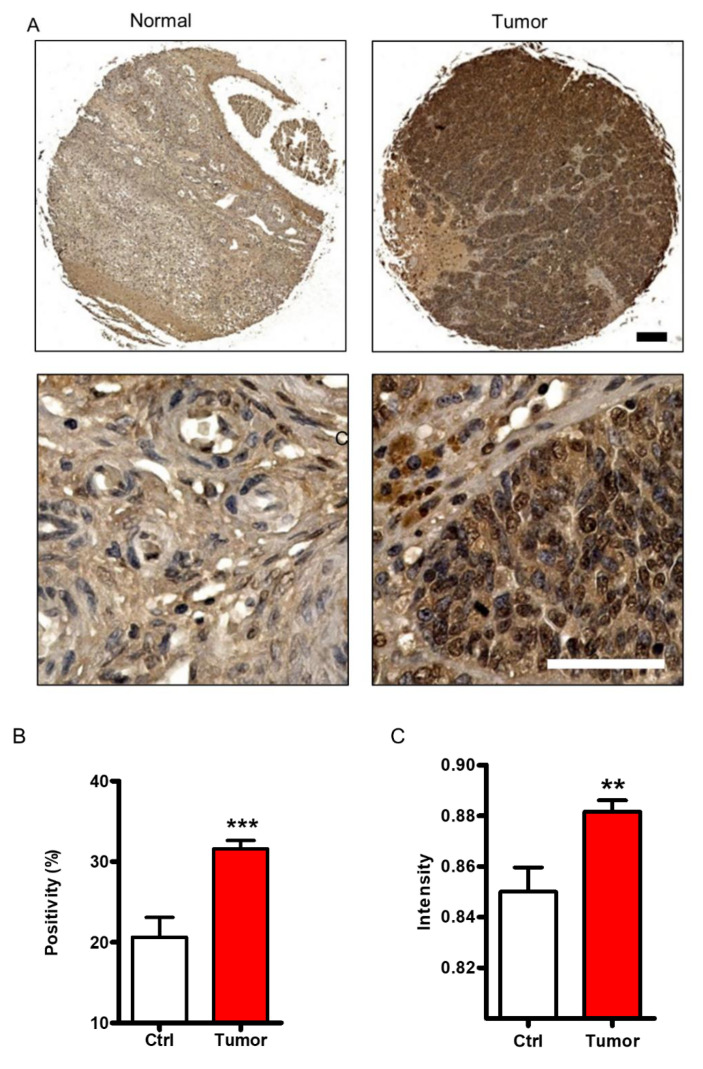
FHL2 protein expression in normal ovarian tissues and EOC tissues. (**A**) Representative images showed FHL2 expression in normal ovarian tissues (left) and epithelial ovarian tissues (right) detected by immunohistochemistry. FHL2 was shown in brown. Nuclei were counterstained with hematoxylin. Scale bar: 150 μm in the upper panel, 50 μm in the lower panel. (**B**) Quantitative data showed the positivity of FHL2 immunosignal in the normal ovarian tissues and epithelial ovarian cancer tissues. *** indicate *p* < 0.001 compared with control (Ctrl). (**C**) Quantitative data showed the immunosignal intensity of FHL2 in the normal ovarian tissues and epithelial ovarian cancer tissues. ** indicate *p* < 0.01 compared with control (Ctrl).

The FHL2 immunosignal was located both in nuclei and cytoplasm of ovarian epithelial cells (Figure 1A). Quantification of the FHL2 immunosignal indicated that the immunosignal positivity, defined as the percentage of FHL2-positive cells relative to the total cells, was significantly increased in the tumor tissues compared with the control tissue (Ctrl) (*p* < 0.001) (Figure 1B). Furthermore, the immunosignal intensity of FHL2 in tumor tissue was significantly higher compared with that of control tissue (*p* < 0.01) (Figure 1C).

### 2.2. Knockdown of FHL2 in EOC Cells Inhibited Cell Growth

Upon confirmation of the FHL2 expression profile among normal and EOC cells, we assessed the expression levels of FHL2 in 1 normal ovarian surface epithelial cells (Hose 969) and six ovarian cancer cell lines, including SKOV-3, IGROV-1, CAOV-3, CAOV-362, COV-644 and A2780.

**Figure 2:**
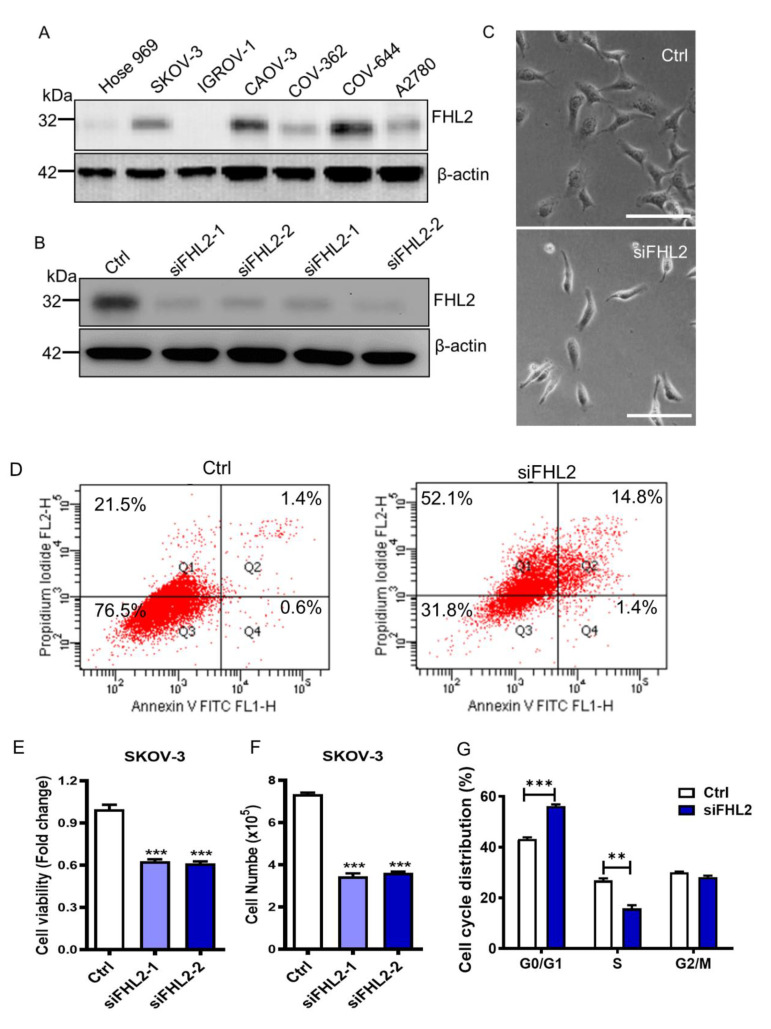
Knockdown of FHL2 inhibits EOC cell growth and induced apoptosis. (**A**) Western blot results showing FHL2 expression levels in Hose 969, SKOV-3, IGROV-1, CAOV-3, COV-362, COV-644 and A2780 cell lines. Beta actin was used as a loading control. (**B**) FHL2 protein levels in SKOV–3 cells were transfected with 2 different FHL2 siRNAs (siFHL2-1, siFHL2-2). SKOV-3 cells transfected with scramble siRNA was used as control. Western blot results showed the FHL2 expression level in SKOV-3 control cells and FHL2 siRNAs knockdown cells. Beta actin was used as a loading control. (**C**) Morphological change of SKOV-3 cells transfected with scramble siRNA (Ctrl) or FHL2 siRNA (siFHL2). Scale bar: 100 μm. (**D**) SKOV-3 control cells and FHL2 knockdown cells (siFHL2) were stain with Annexin V-FITC/PI dual staining kit, and cell apoptosis was detected by the flow cytometry. Q1, Q2, Q3 and Q4 indicate proportion of dead cells, early apoptotic, live, and late apoptotic cells, respectively. Experiments were repeated at least 3 times, and representative images were shown. (**E**) MTT method results showed the cell viability changes in SKOV-3 control cells (Ctrl) and FHL2 knockdown cells transfected with 2 different siRNAs (siFHL2-1, siFHL2-2). Each bar represents mean ± S.E.M. (n = 3). (**F**) Cell number were quantified to show the FHL2 knockdown effect on SKOV-3 control cells (Ctrl) and FHL2 knockdown cells transfected with 2 different siRNAs (siFHL2-1, siFHL2-2). (**G**) Cell cycle distribution in SKOV–3 control (Ctrl) and FHL2 knockdown cells (siFHL2). Control and treated cells were labeled with propidium iodide and flow cytometry was used to determine the cell-cycle distribution. Each bar represents mean ± S.E.M. (n = 3). ** indicated *p* < 0.01 compared with control (Ctrl), *** indicated *p* < 0.001 compared with control (Ctrl).

As shown in Figure 2A, FHL2 more highly expressed in 5 EOC cells (except IGROV-1) compared to the normal epithelial cells Hose 969, indicating that the FHL2 was highly activated in these ovarian cancer cell lines (Figure 2A). Among the six epithelial ovarian cancer cell lines, FHL2 had relatively higher expression levels in SKOV-3, CAOV-3 and COV-644 cells, and lower expression in IGROV-1 cells. Considering SKOV-3 is widely used in many EOC studies, and has the ability to form xenografts in vivo [20], we employed SKOV-3 cells as a cell model to evaluate the knockdown effect of FHL2, we also employed IGROV–1 cells to identify the effects of overexpression of FHL2 on EOC cells.

In order to directly assess the effects of FHL2 on the SKOV-3 cell growth, we examined whether knockdown of FHL2 could alter the proliferation of the SKOV-3 cells. We used two different FHL2-siRNAs to reduce the FHL2 expression. Western blotting results showed the ideal inhibition efficiencies in response to both of the FHL2 siRNAs (siFHL2-1, siFHL2-2) when compared with the control cells transfected with scrambled siRNA (Ctrl) (Figure 2B). Interestingly, knockdown of FHL2 induced dramatic morphological changes of SKOV-3 (Figure 2C) characterized by a skinny and stress fiber cellular morphology, which was related with a pro-apoptotic phenotype when compared with the cells transfected with scrambled siRNA (Figure 2C). Annexin V-FITC apoptosis assays confirmed that knockdown FHL2 significantly increased SKOV-3 cell apoptosis compared with the control (Figure 2D). MTT assays revealed that silencing FHL2 significantly reduced SKOV3 cell viability compared with the control group (Figure 2E). Consistently, cell numbers of FHL2 knockdown groups were significantly lower than control group (Figure 2F). Knockdown of FHL2 also significantly blocked SKOV-3 cell cycle progression (Figure 2G). Taken together these results indicated that knockdown of FHL2 in EOC cells inhibited cell growth and induced cell apoptosis.

### 2.3. Ectopic Expression of FHL2 Improve EOC Cell Growth

**Figure 3:**
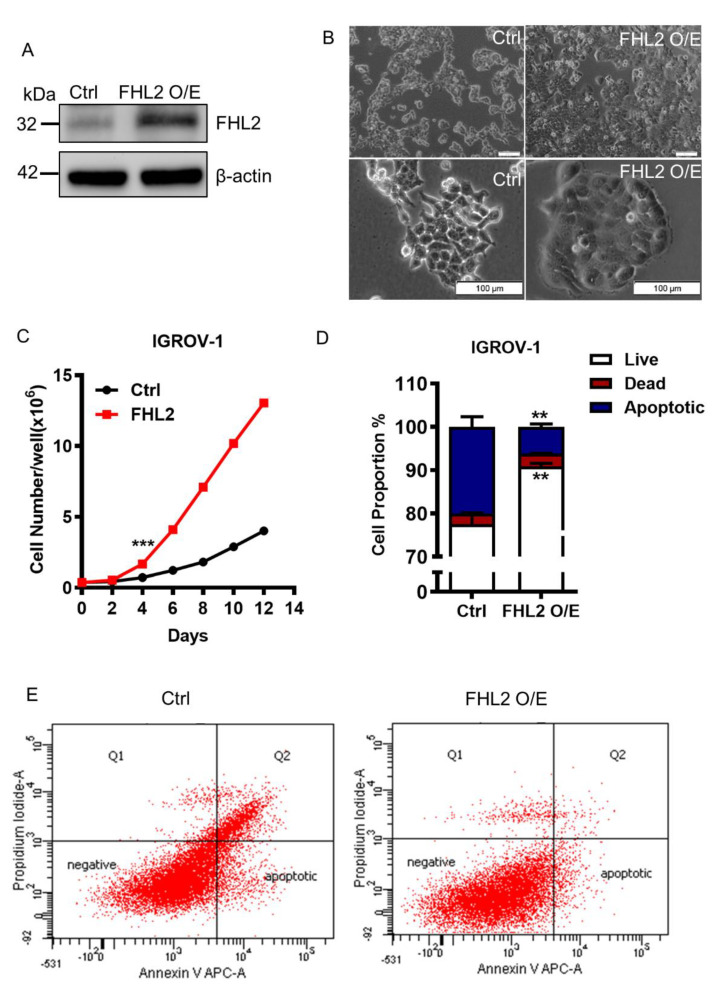
Ectopic expression of FHL2 promote EOC cell growth and induced cell morphological change. (**A**) IGROV-1 cells were transfected with letenvirus-based control vectors (Ctrl) or FHL2 overexpression vectors (FHL2 O/E) Beta actin was used as a loading control. (**B**) Representative images showed the morphological change of IGROV-1 control cells (Ctrl) and FHL2 overexpressing cells (FHL2 O/E). Scale bar: 100 μm. (**C**) Growth curve of IGROV-1 control cells (Ctrl) and FHL2 overexpressing cells (FHL2). Each point represents mean ± S.E.M. *** indicate *p* < 0.001. (**D**) Quantification results showed the apoptotic, live and dead cell proportion in IGROV-1 control (Ctrl) and FHL2 overexpression cells (FHL2 O/E). (**E**) IGROV-1 control (Ctrl) and FHL2 overexpression cells (FHL2 O/E) cells were cultured in serum reduced (2%) culture medium. Cells then stained with Annexin V-APC/PI dual staining kit for flowcytometry analysis.

To gain a better understanding of FHL2 function in epidermal ovarian cancer cell growth, we transfected IGROV-1 cells, which express relatively low endogenous FHL2 level, with a lentivirus based FHL2 expression vector (FHL2 O/E) or an empty control vector (Ctrl). The western blotting result revealed that FHL2 was successfully overexpressed in IGROV–1 transfected cells (Figure 3A). Notably, we observed that FHL2 overexpression IGROV–1 cells induced irregular morphological change. The cell boundary almost disappeared compared with the control group (Figure 3B).

The IGROV-1 cells overexpressing FHL2 grew faster than control cells (Figure 3C). The cell number was significantly increased in the FHL2 overexpression cells 4 days after culture (Figure 3C). The IGROV-1 cells with ectopic expression of FHL2 reached confluence earlier than control cells and maintained a higher growth rate after confluence (Figure 3B, upper panel; Figure 3C). These results showed that ectopic expression of FHL2 significantly promoted IGROV-1 cell growth. The Annexin V/FITC apoptosis assays elucidated that overexpression of FHL2 successfully reduced IGORV-1 cell apoptosis in a reduced serum culture system (Figure 3D,E), indicating that ectopic expression of FHL2 can stimulate EOC cell growth and inhibit cell apoptosis.

### 2.4. FHL2 Regulate AKT Expression in EOC Cells

**Figure 4:**
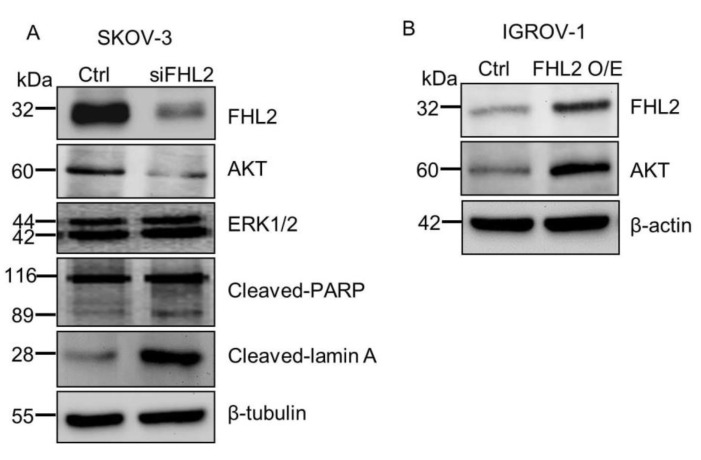
FHL2 regulate AKT expression in EOC cells. (**A**) Western blot results showed FHL2, AKT and apoptosis related protein levels in SKOV-3 control and FHL2 knockdown cells (siFHL2). Beta tubulin was used as loading control. (**B**) Western blot results showed FHL2 and AKT expression level in IGROV-1 control and FHL2 overexpression cells (FHL2). Beta actin was used as loading control.

Mechanistically, we found that knockdown of FHL2 in SKOV-3 cells significantly decreased expression of AKT (Figure 4A), a critical regulator of cell survival and viability [21]. ERK1/2 expression remained unchanged. Consistently, ectopic expression of FHL2 in IGROV-1 cells significantly increased AKT protein (Figure 4B). Besides, knockdown of FHL2 induced the apoptosis marker expression, such as cleaved-PARP and cleaved-lamin A, which are consistently with the apoptotic phenotype in SKOV-3 FHL2 knock down cells. These results showed that FHL2 may regulate EOC cells survival by regulating AKT expression.

### 2.5. FHL2 Enhance Anchorage-Independent EOC Cell Growth

**Figure 5:**
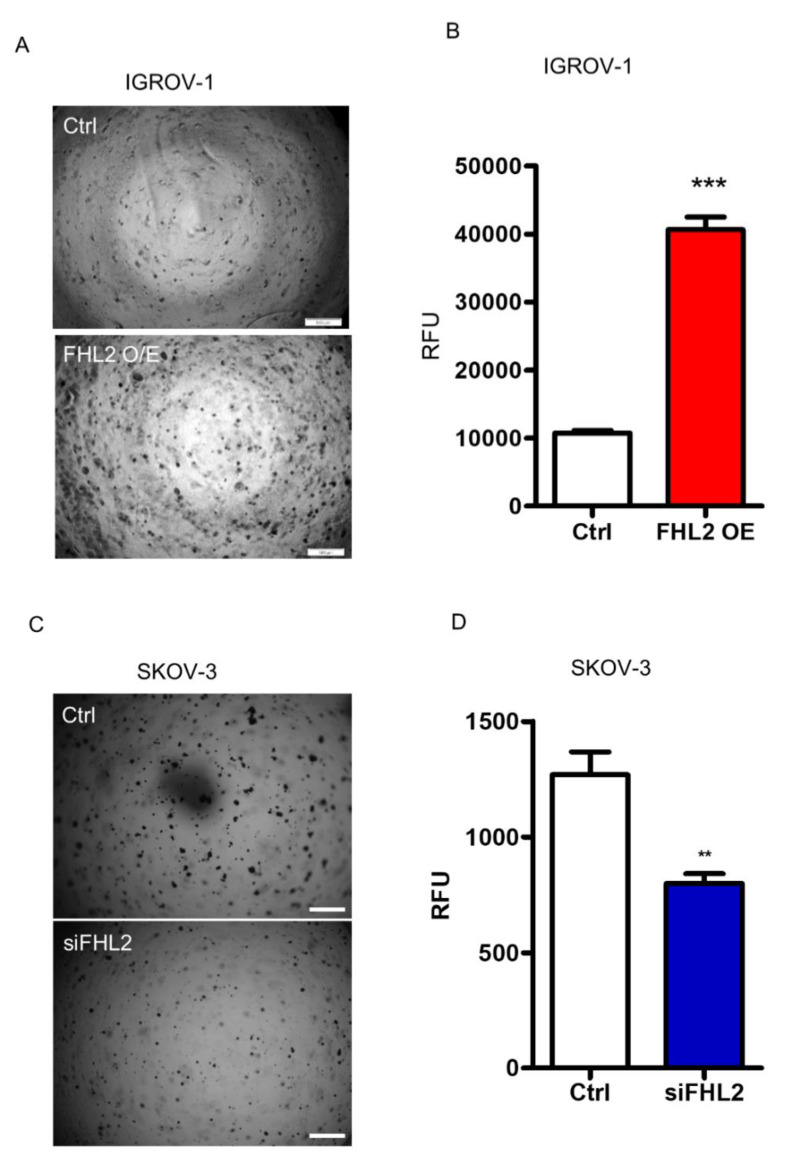
FHL2 regulated anchorage-independent growth of EOC cells. (**A**) Representative images showed colony formation by IGROV-1 control (Ctrl) and stable FHL2 overexpression cells (FHL2 O/E). (**B**) Quantitative analysis showed the differences of anchorage-independent growth of IGROV-1 control and FHL2 overexpression cells based on the fluorescence detection. (**C**) Representative images showing colony formation by SKOV-3 control (Ctrl) and FHL2 knockdown cells (siFHL2). Scale bar: 500 μm. (**D**) Quantitative analysis showed the differences of anchorage-independent growth of SKOV-3 control (Ctrl) and FHL2 knockdown cells (siFHL2) based on the fluorescence detection.

To determine the tumorigenicity of FHL2 in vitro, the soft agar assay was performed in both SKOV-3 and IGROV-1 cells. IGROV-1 control cells with low FHL2 expression level formed a few tiny colonies on the soft agar. On the contrary, the IGROV-1 FHL2 overexpression cells formed more and larger colonies in the soft agar (Figure 5A). A fluorescence-based cell transformation assay was also used to confirm the anchorage-independent cell growth. The results confirmed that overexpression of FHL2 in IGROV-1 cells stimulates colony formation, which was indicated by a significant increase (*p* < 0.001) in relative fluorescence units (RFU) (Figure 5B).

Consistently, in the SKOV-3 cells which express relatively high endogenous FHL2 levels, the control cells formed visible large colonies. However, SKOV-3 cells transfected with FHL2 siRNA, formed less and smaller colonies in soft agar (Figure 5C). Quantitative analysis showed that the knockdown of FHL2 significantly inhibited anchorage-independent growth of SKOV-3 cells as indicated by lower RFU in knockdown cells (*p* < 0.01, Figure 5D). These data clearly show that FHL2 regulates EOC cell anchorage-independent growth in vitro.

### 2.6. FHL2 Regulates EOC Cell Migration and Invasion In Vitro

**Figure 6:**
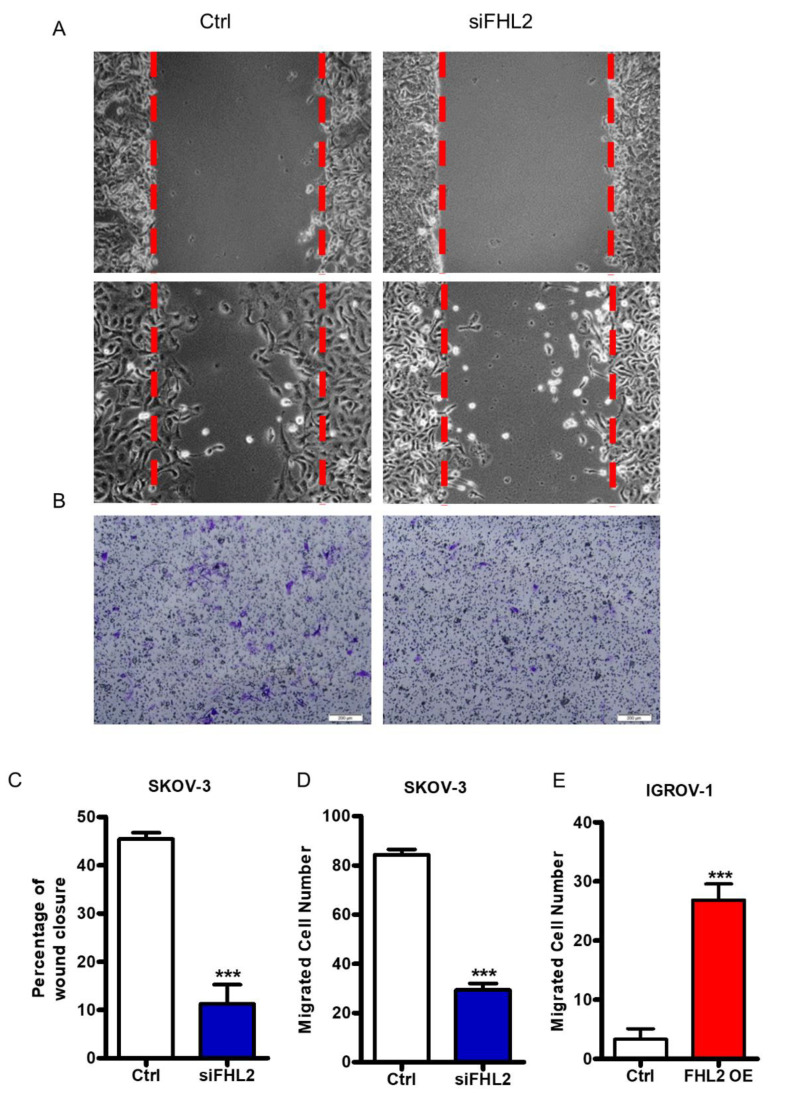
FHL2 regulates EOC cells migration and invasion. (**A**) Wound healing assay was performed to determine the effect of FHL2 on SKOV-3 cell migration. Representative images showed the migration of control cells (Ctrl, left panel) and FHL2 knockdown cells (siFHL2, right panel). Cells were allowed to migrate for 15 h before wound closure area measured. (**B**) Cell migration were analyzed by transwell migration assay. Representative images showed the migration of SKOV–3 control cells (left) and FHL2 knockdown cells (right). Scale bar: 200 μm. (**C**) The wound area was quantified with MicrosuitTM FIVE software. Each bar represents mean ± SEM. ***: *p* < 0.001 compared with control (Ctrl). (**D**) Migrated cells were counted manually under a microscope. Each bar represents mean ± SEM. ***: *p* < 0.001, compared with control. (**E**) Cell migration were evaluated by transwell assay in IGROV-1 control (Ctrl) and IGROV–1 FHL2 overexpression cells (FHL2 O/E). Each bar represents mean ± SEM. ***: *p* < 0.001.

**Figure A1:**
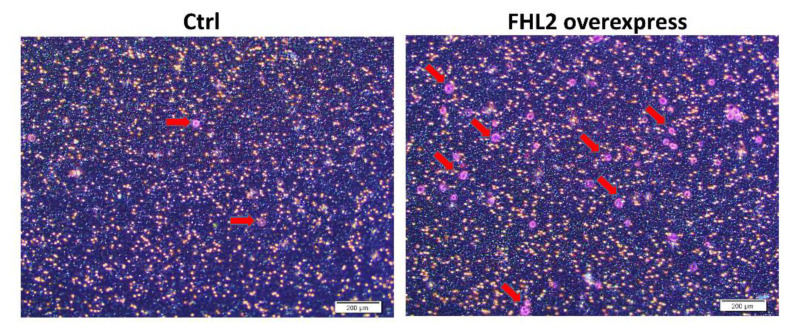
FHL2 overexpression promote IGROV-1 cells migration by transwell assay. Scale bar: 200 μm. Red arrow indicate the migrated cells.

Cancer metastasis and failures to clinically treat metastases are responsible for the majority of patient deaths from solid tumors [22]. Cell migration is a pivotal step in the metastatic process. SKOV–3 represent a metastatic EOC cell line. To evaluate the FHL2 regulation on EOC cell migration, wound healing assay and transwell assay were performed. Wound healing assay results showed that knockdown of FHL2 inhibited SKOV-3 cell migration (Figure 6A,C). Consistently, transwell assay results also showed that the number of migrated cells were significantly decreased in the FHL2 knockdown cells compared with control cells (Figure 6B,D). Due to the special growth pattern, wound healing assay could not be performed in IGROV-1 cells. However, transwell assay results showed that IGROV-1 cells with stable ectopic expression of FHL2 (FHL2 OE) exhibited greater cell migration compared with control cells ( Figure 6E and Figure A1). These results indicated that FHL2 can regulate EOC cells migration in vitro.

### 2.7. Knockdown of FHL2 Inhibited Tumorigenesis In Vivo

**Figure 7:**
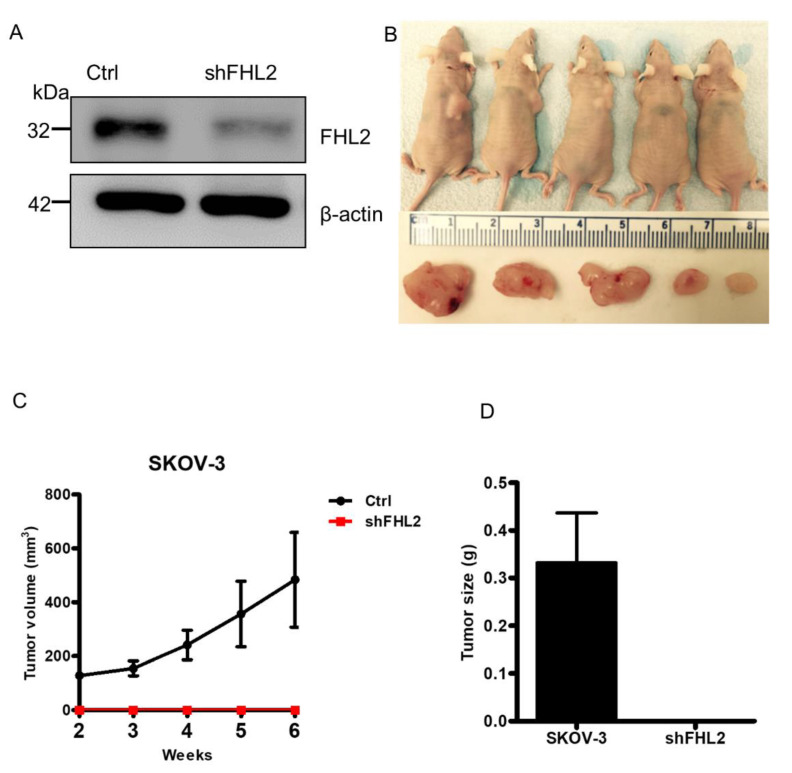
Knockdown of FHL2 inhibited EOC tumorigenesis in vivo. (**A**) SKOV-3 cells were transfected by either lentivirus-based non-targeting (Ctrl) or FHL2 shRNA vectors (shFHL2), and stable cell lines were constructed. Western blot results showed FHL2 expression in control (Ctrl) and FHL2 knockdown stable cells (shFHL2). Beta actin was used as loading control. (**B**) SKOV-3 cells transfected with lentivirus-based non-targeting or FHL2 knockdown vector (shFHL2) were implanted subcutaneously into the right and left shoulders of athymic nude mice, respectively. Images showed the tumor formation in the right shoulders of nude mice (Top panel). The formed tumors from control were isolated and presented (lower panel). No xenograft was found in FHL2 knockdown group. The minimum scale of the ruler is mm. (**C**) Growth curve showed the xenografts derived from control and FHL2 knockdown cells (shFHL2). Each point represents mean tumor volume ± S.E.M. of 5 mice. (**D**) Weights of tumor xenografts derived from control and FHL2 knockdown group (shFHL2). Each bar represents mean ± S.E.M.3.

**Figure A2:**
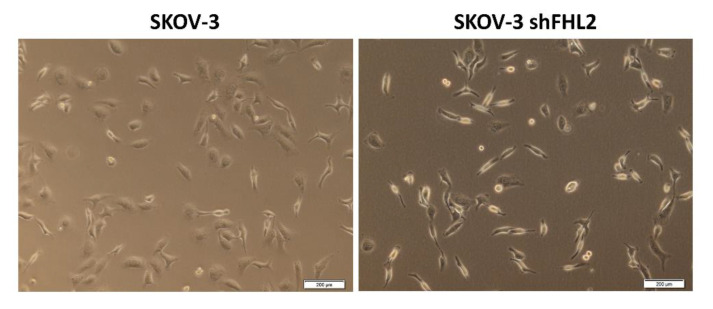
Representative images showed the morphological change in SKOV-3 control and stable FHL2 knockdown cells (shFHL2). Scale bar: 200 μm.

To confirm that FHL2 may regulate tumorigenesis in vivo, lentivirus-based FHL2 shRNA was used to knock down FHL2 protein in SKOV-3 cells. And lentivirus-based non-targeting shRNA was used as control (Ctrl). The knockdown of FHL2 was validated by western blot (Figure 7A). The SKOV-3 FHL2 stable knockdown cells also showed the similar morphological change as found in SKOV-3 cells transfected with FHL2 siRNA (Figure A2). Then FHL2 knock down cells and control cells were implanted subcutaneously into the left and right shoulders of athymic nude mice, respectively. As reported, SKOV-3 control cells formed tumors in 100% (5/5) at the right shoulder of athymic nude mice within 2 weeks after cell injection (Figure 7C). The xenograft tumors developed rapidly after third weeks following injection (Figure 7C). However, injection of the SKOV–3 shFHL2 cells into the nude mice did not induce any visible xenograft at the left shoulder (Figure 7B–D). These results showed that FHL2 is critical for the initiation of EOC tumorigenesis.

## 3. Discussion

The average lifetime risk of developing ovarian cancer is 1.3%, the equivalent of 1 in 78 women [1]. Our previous study showed that FHL2 can regulate the ovarian cancer development and progression, for example, FHL2 acts as a GCT tumor cell growth-promoting factor [18]. Overexpression of FHL2 protein in EOC has been reported [19], however, the role of FHL2 in EOC initiation and progression is unknown. In the present study, we confirmed that FHL2 protein is elevated in EOC tissues compared with normal ovary. By knockdown FHL2 in SKOV-3 (with relatively high endogenous expression level) and/or overexpressing FHL2 in IGROV-1 (with relatively lower endogenous expression level) cells, we found that FHL2 may play an important role in the initiation and progression of EOC.

In a previous study, FHL2 was found to be expressed in 13 epithelial ovarian carcinomas and 6 nonneoplastic tissues from the human postmenopausal ovary [19]. However, the quantitative evaluation of FHL2 expression level was not mentioned. Here, we further evaluated FHL2 expression in 78 EOC tissues. These results showed a strong evidence that FHL2 may be a potential diagnosis biomarker of EOC [23].

Researches on FHL2 has focused on examining its role in tumor cells growth and metastasis. In MDA-MB 231 breast cancer cell lines, overexpression FHL2 promoted cancer development by mediating transcriptional activation of MAPK target genes [24]. And in colorectal cancer, knockdown FHL2 repressed the FOXK1-dependent cell growth and metastasis [25]. Consistently, we revealed that knockdown of FHL2 in SKOV-3 cells resulted in significantly reduced proliferation, enhanced apoptosis in vitro. Most importantly, knockdown of FHL2 blocked the EOC carcinogenesis in vivo. We also found that overexpression of FHL2 in IGROV-1 cell increased cell proliferation, migration and repress apoptosis in vitro. Previous studies have found FHL2 can regulate cell cycle progression via controlling of cyclin D1 expression [26,27]. We found that ectopic expression of FHL2 reduced IGROV–1 cell apoptosis, suggesting that FHL2 may improve EOC cell viability by inhibiting cell apoptosis. These findings indicate that FHL2 could be a treatment target for EOC. However, more research is needed to understand the role of FHL2 in other cancers.

Cell migration and invasion are pivotal steps in the metastatic process [28]. Several studies have reported the FHL2 regulates migration and contraction during wound healing via binding with actin in order to modulate cytoskeleton dynamics [29,30]. Consistently, our transwell and wound healing results illuminated that overexpression FHL2 can promote EOC cell migration and invasion while transfected with siFHL2 strongly suppressed SKOV-3 migration. These data implied that FHL2 may stimulate EOC metastasis progress [31,32,33]. Interestingly, our data also indicate that FHL2 could regulate the AKT expression, suggesting FHL2 may regulate EOC development and progression via AKT signaling pathway. Recent researches demonstrated that knockdown of FHL2 stimulated either cleaved-PARP or cleaved-lamin A, which are critical for nuclear stability and chromatin structure [34,35]. Consistently, our data also demonstrated the knockdown of FHL2 increased levels of these pro-apoptotic markers. Nonetheless, the precise mechanism and signaling pathway of FHL2 in EOC require further exploration.

In summary, our study shows that FHL2 is overexpressed in EOC tissues. Overexpression of FHL2 promote proliferation, reduce apoptosis and drive migration. The mechanism of action by which FHL2 regulated EOC cell proliferation and apoptosis possibly involved AKT pathway. The findings in our study indicated that FHL2 may serve as a molecular target for therapeutic drug development against EOC.

## 4. Materials and Methods

### 4.1. Cell Lines and Human EOC Tissue Slides

SKOV-3 and CAOV-3 cell lines were commercially purchased from American Type Culture Collection (Manassas, VA, USA). COV-362, COV-644 and A2780 cell lines were obtained from the European Collection of Authenticated Cell Cultures (ECACC). Hose 969, and IGROV-1 cell lines were kindly gifted by Bo R. Reuda (Vincent Center for Reproductive Biology, Massachusetts General Hospital, Boston, MA, USA). Cell lines were validated for their authenticity with short tandem repeat (STR) analysis performed by both the Riken Biosource Center (Riken Cell Bank, Tsukuba, Ibaraki, Japan) and the Genetica DNA Laboratories (Burlington, NC, USA). The cells were incubated in DMEM (Life Technologies, Inc., Gaithersburg, MD, USA) supplemented with 10% fetal bovine serum, 100 μg/mL streptomycin and 100 units/ml penicillin (Life Technologies, Inc., Gaithersburg, MD, USA) in a humidified incubator at 37 °C with an atmosphere of 5% CO2. Human normal and EOC tissue were purchased from US Biomax (Rockville, MD, USA).

### 4.2. Immunohistochemistry Staining

To examine the expression of FHL2 in EOC tissues, we performed the peroxidase-based immunohistochemistry as previously described [36]. Tissue array was commercially purchased from US Biomax Inc. Sections were scanned using an iSCAN Coreo Slide Scaner (Ventana Medical Systems, Inc., Oro Valley, AZ, USA). The intensity of the positive signal was quantified and recorded using Aperio ImageScope software (Aperio Technologies, Inc., Vista, CA, USA).

### 4.3. Western Blot Analysis

Cells were lysed with ice-cold cell lysis buffer, and supernatants were harvested. Protein levels were determined using Western blot with a protocol as previously mentioned [18]. The immunosignal was detected with a Super Signal West Femto Chemiluminescent Substrate Kit (Pierce/Thermo Scientific, Rockford, IL, USA). The images were captured and analyzed with a UVP gel documentation system (UVP; LLC, Upland, CA, USA).

### 4.4. Cell Proliferation Analysis

To determine the effect of FHL2 on EOC cell proliferation, SKOV-3 and IGROV-1 cells were plated in 6 well plates and cultured in a growth medium supplemented with 5% FBS until 60% confluent. The SKOV-3 cells were then transfected with non-targeting scramble siRNA or FHL2 siRNA for 6 h FHL2 siRNA and a scrambled siRNA were purchased from Darmacon ThermoFisher (Waltham, MA, USA). The cells were trypsinized after siRNA transfection 72 h for counting cell numbers. The effect of FHL2 on EOC cell proliferation was also determined in IGROV-1 control cells that FHL2 overexpression cells. Each cell line was cultured in the growth medium containing 10% FBS for up to 12 days. Cell number were quantified every other day with a Countess Automated cell counter (Invitrogen, Carlsbad, CA, USA).

### 4.5. Cell Viability Analysis

Cells were plated in 6-well plates and transfected with siRNA or scrambled siRNA, control vectors, FHL2—expressing vectors for 48 h before performing the 3-(4,5-dimethylthiazol-2-yl)-2,5-diphenyltetrazolium bromide (MTT) assay using a Vybrant MTT Assay Kit (Life Technologies, Carlsbad, CA, USA) according to the manufacturer’s instructions.

### 4.6. Cell Cycle and Apoptosis Analysis

Cell cycle and apoptosis analysis were performed by flow cytometry. Treated cells were trypsinized, harvested, fixed and permeabilized with 70% ethanol for overnight. The cells were then resuspended with propidium iodide buffer for 30 min at 37 °C and flow cytometry was used to determine the cell-cycle distribution. Apoptosis was analyzed by cell surface presence of Annexin V using the Annexin V-FITC/PI or Annexin V-APC/PI Dual Staining Apoptosis Assay Kit as described by the manufacturer (BioVision, Inc., Milpitas, CA, USA).

### 4.7. Cell Migration Assays

A wound-healing assay was used to examine the FHL2 function on EOC cell migration. The ‘wound’ area was measured using computerized Microsuite FIVE imaging software (Olympus America, Inc., Center Valley, PA, USA) [37]. Furthermore, we also performed a transwell cell migration assay established in our laboratory to confirm the effect of FHL2 on EOC migration [38]. Experiments were repeated in triplicate, and at least three inserts were used for each treatment group.

### 4.8. Colony Formation Assay

The effect of FHL2 on the transformation and anchorage independent growth of EOC was performed with a Cytoselect 96-Well Cell Transformation assay kit (Cell Biolabs, Inc., San Diego, CA, USA) according to the manufacturer’s instructions. This soft agar-based cell colony formation kit was also used to determine the effect of FHL2 on the anchorage-independent growth of EOC in vitro.

### 4.9. Establishment of FHL2 Knockdown and Overexpression Stable Cell Lines

ShFHL2 and FHL2 overexpression lentivirus-based vectors, and non-targeting control vector were commercially purchased from Applied Biological Materials, Inc. (Richmond, BC, Canada). To construct FHL2 stable knock out cell line, SKOV-3 were cultured to 30–40% confluence and then transfected with either lentivirus-based shFHL2, or control vectors, respectively. SKOV-3 cells were then selected for 1 week in growth medium with 4 μg/mL puromycin. The FHL2 knockdown efficiency was measured by western blot.

To construct stable FHL2 overexpression cells, IGROV-1 cells were cultured to 40–50% confluence and then were transfected with lentivirus-based human FHL2 expression and control constructs. Two days following transfection, cells were selected with puromycin (3–5 ug/mL) for 7 days. FHL2 expression level in stable cell lines were examined by western blot.

### 4.10. Tumorigenicity in Nude Mice

FHL2 regulatory effects on tumor growth was evaluated in a nude mouse xenograft model. The use of animal was approved by the Institutional Animal Care and Use Committee (IACUC) at the University of Nebraska Medical Center. Control SKOV-3 cells and SKOV-3 cells with stable knockdown of FHL2 were cultured, trypsinized and collected. Cell suspensions (5 million cells/100 μL of DMEM) were mixed with 100 μL of Matrigel BD Bioscience (San Jose, CA, USA) and inoculated subcutaneously into the shoulders (left side and right side) of 5-week old female athymic nude mice (Harlan Sprague Dawley). Mice were maintained under a 12 h light/12 h dark cycle with free access to water and standard mouse diet. At the end of the experiment, the mice were sacrificed. Tumors were dissected and weighted. Tumor size was measured 6 weeks after initial inoculation. The volumes of tumors were calculated as follows: V = R12 × R22 × 3.142/6, where R1 and R2 are the short and long diameters of the tumors, respectively.

### 4.11. Statistical Analysis

All experiments were repeated at least three times unless otherwise noted. 5 mice were used in the in vivo animal studies. Statistical analysis was conducted using GraphPad Prism software (GraphPad Software, Inc., La Jolla, CA, USA). Data were analyzed for significance of difference by one-way analysis of variance with Tukey’s post-test (multiple groups) or Welch’s *t*-test (two groups). A *p*-value of 0.05 was considered to be significant.
